# Pre-consultation educational group intervention to improve shared decision-making in postmastectomy breast reconstruction: study protocol for a pilot randomized controlled trial

**DOI:** 10.1186/1745-6215-14-199

**Published:** 2013-07-06

**Authors:** Jennica Platt, Nancy Baxter, Jennifer Jones, Kelly Metcalfe, Natalie Causarano, Stefan OP Hofer, Anne ONeill, Terry Cheng, Elizabeth Starenkyj, Toni Zhong

**Affiliations:** 1 UHN Breast Restoration Program, Division of Plastic and Reconstructive Surgery, 8N 871, 200 Elizabeth Street, Toronto, ON M5G 2C4, Canada

## Abstract

**Background:**

The Pre-Consultation Educational Group Intervention pilot study seeks to assess the feasibility and inform the optimal design for a definitive randomized controlled trial that aims to improve the quality of decision-making in postmastectomy breast reconstruction patients.

**Methods/design:**

This is a mixed-methods pilot feasibility randomized controlled trial that will follow a single-center, 1:1 allocation, two-arm parallel group superiority design.

Setting: The University Health Network, a tertiary care cancer center in Toronto, Canada.

Participants: Adult women referred to one of three plastic and reconstructive surgeons for delayed breast reconstruction or prophylactic mastectomy with immediate breast reconstruction.

Intervention: We designed a multi-disciplinary educational group workshop that incorporates the key components of shared decision-making, decision-support, and psychosocial support for cancer survivors prior to the initial surgical consult. The intervention consists of didactic lectures by a plastic surgeon and nurse specialist on breast reconstruction choices, pre- and postoperative care; a value-clarification exercise led by a social worker; and discussions with a breast reconstruction patient.

Control: Usual care includes access to an informational booklet, website, and patient volunteer if desired.

Outcomes: Expected pilot outcomes include feasibility, recruitment, and retention targets. Acceptability of intervention and full trial outcomes will be established through qualitative interviews. Trial outcomes will include decision-quality measures, patient-reported outcomes, and service outcomes, and the treatment effect estimate and variability will be used to inform the sample size calculation for a full trial.

**Discussion:**

Our pilot study seeks to identify the (1) feasibility, acceptability, and design of a definitive RCT and (2) the optimal content and delivery of our proposed educational group intervention. Thirty patients have been recruited to date (8 April 2013), of whom 15 have been randomized to one of three decision support workshops. The trial will close as planned in May 2013.

**Trial registration:**

NCT01857882

## Background

Women with a history of breast cancer now constitute the largest group of cancer survivors [1], with over 85% surviving for greater than 5 years after diagnosis [2]. Mastectomy remains a common form of breast cancer treatment, with 37% of American women undergoing mastectomy as their definitive cancer treatment in 2006 [3]. The goal of breast reconstruction is to reconstruct a mastectomy defect without affecting cancer outcomes [4]. The opportunity to restore the breast mound through reconstructive surgery has been shown to enhance a woman’s self-image and femininity [5-7], as well as provide psychosocial benefit [8].

The majority of women who undergo breast reconstruction are satisfied with the outcome and the aesthetic result [5,9-11]. However, one fourth of women report being dissatisfied with some component of their cancer or reconstructive care [12]. Failure of the physician to provide adequate information about treatment options is the most frequent source of cancer patient dissatisfaction [13], and breast reconstruction patients have expressed a need for further information regarding the complex decision to pursue breast reconstruction [14-16]. Information dissatisfaction and a mismatch between a patients’ preferred and actual role in decision-making may contribute to decision regret in breast oncology patients [12,17-19]. Implementation of strategies to incorporate shared decision-making can improve the quality of treatment decisions [20]. Developing the self-confidence to make and express a treatment decision (defined as decision self-efficacy [21]) is a necessary component of shared-decision making, and this attribute can be enhanced through decision support interventions [22].

**Figure 1:**
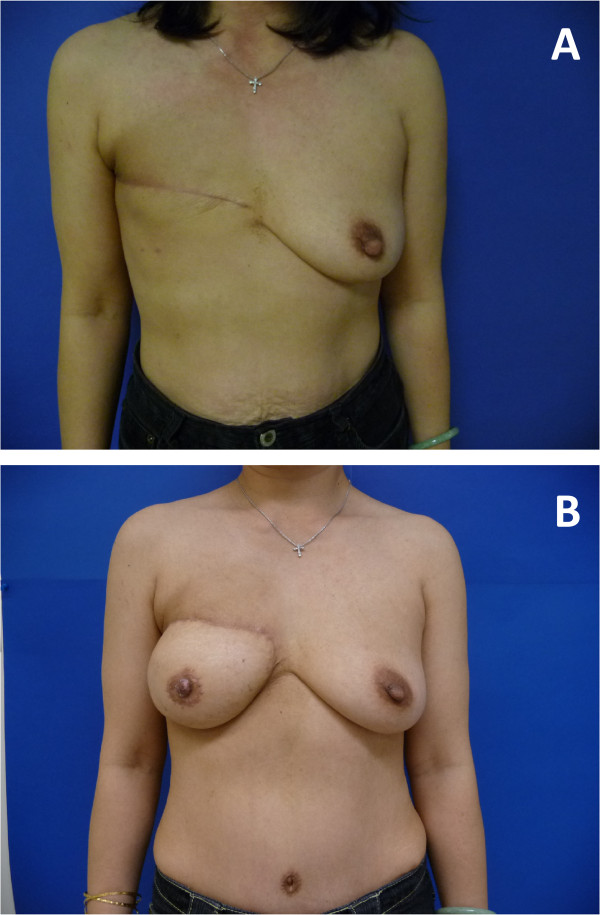
**Delayed breast reconstruction. (A)** Preoperative photograph demonstrating right mastectomy defect after post-mastectomy radiation. **(B)** Postoperative photograph after delayed breast reconstruction using autologous tissue reconstruction.

Given the financial and time constraints that exist in our current health-care system, it is not feasible to relay detailed information regarding every aspect of breast reconstruction during a single patient consultation. In addition, the breast reconstruction discussions can be highly complex, as there are many different techniques (implant vs. autologous tissue, one-stage vs. two-stage), timing (delayed vs. immediate), and complications that are unique to each procedure [4,23]. In such scenarios of complex medical decision-making, decision support techniques may be an effective solution to information provision [13] and shared decision-making [14,24]. As a result, we developed a pre-consultation educational group intervention delivered in a group setting for women considering breast reconstruction, with the aims to fill an existing information-gap, promote high-quality decision-making, and enhance decision self-efficacy. This pilot study will be the first step in the evaluation of our educational group intervention, and the results will be used to determine the feasibility and inform the optimal design for a definitive randomized controlled trial. Figure 1 shows a pre- and postoperative photo of a patient who underwent delayed breast reconstruction using autologous tissue reconstruction.

### Hypothesis

Patients seeking consultation for breast reconstruction allocated to the pre-consultation educational group intervention plus usual care will have greater decision self-efficacy, satisfaction with information, decision preference, and decision choice, perceived involvement in care, breast reconstruction knowledge, and less decisional conflict as measured approximately 1 week after intervention and surgical consultation compared with patients allocated to receive usual care alone.

### Pilot study objectives

1. To determine feasibility and acceptability of randomization, intervention uptake, and data collection

2. To assess implementation and fidelity of the outcome measures and intervention

3. To pilot trial procedures including recruitment (giving information and obtaining preliminary consent via telephone recruitment), randomization, intervention delivery (workshop scheduled same day as initial surgical consult), and outcome measurement (mailed questionnaires)

4. To use qualitative research methods to assess the content and acceptability of the intervention and learn from patient interviews how to improve the delivery of the intervention for the full trial

5. To obtain estimates of variance and a preliminary estimate of the effect of the intervention on preliminary trial outcomes (decision self-efficacy, satisfaction with information, decision preference and decision choice, decisional conflict) and service outcomes such as duration of consultation.

## Methods/design

This is a mixed-methods pilot feasibility randomized controlled trial that will follow a single-center, 1:1 allocation, two-arm parallel group superiority design. The study setting will be the University Health Network (UHN), a tertiary care cancer center in Toronto, Canada, with three plastic and reconstructive surgeons who specialize in breast reconstruction. Research ethics board approval was obtained from the University Health Network (11-1027-CE).

### Participants

**Figure 2:**
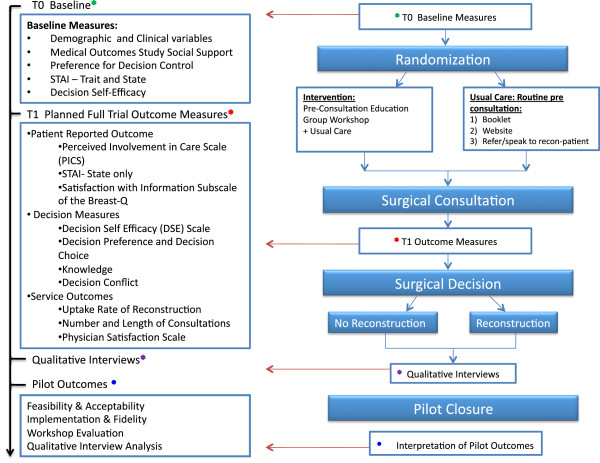
Study design flowchart and participant timeline.

Adult women referred to one of the plastic surgeons for consideration of breast reconstruction will be eligible to participate. The study coordinator is responsible for identification and recruitment of potentially eligible participants. Because the intervention (educational workshop) will occur *before* the initial surgical consultation, it is necessary to identify subjects from faxed referrals and determine their eligibility via telephone confirmation at the time of recruitment and prior to the initial surgical consultation. Participants will be excluded if they are referred for reconstruction after atypical breast malignancy (e.g., angiosarcoma) or metastatic breast cancer, secondary breast reconstruction, have cognitive impairment or uncontrolled psychiatric diagnosis, or cannot read or write in English. Once eligibility has been confirmed with the study coordinator, subjects who indicate an interest in participation will be enrolled after providing informed consent over the telephone, and study packages will subsequently be mailed. Participants will be officially registered upon receipt of signed consent through return mail. Baseline measures (T0) will be assessed prior to randomization and returned in postage-paid envelopes. Participants in the study and control groups will complete T1 measures approximately 1 week after the initial surgical consultation. The participant timeline is outlined in Figure 2.

### Randomization and allocation of interventions

Once informed consent has been obtained from each participant and the baseline questionnaire returned by mail, the next opaque, sealed envelope in sequence will be opened by the study coordinator to determine the participant’s randomized treatment allocation. A computer-generated random allocation sequence will be created and sealed in opaque envelopes by the program biostatistician independent form the study coordinator with 1:1 allocation to the educational group intervention or usual care and balanced in blocks of ten.

### Intervention

Patients in the experimental group will participate in a pre-consultation educational group intervention in addition to receiving usual care. The intervention will be 2 h in duration on the morning of the consultation and will be facilitated by a dedicated social worker from psycho-oncology. Concerning the workshop development and structure, the intervention will incorporate the key components of shared decision-making (patient-physician involvement, shared information, expression of preferences) [13] and decision support (information provision, values clarification and patient involvement) [25-27] with the philosophy of delivering supportive care to cancer patients [28-30].

•Surgeon – 30 min: treatment options for breast reconstruction with indications/contraindications, advantages/disadvantages, expected postoperative course, aesthetic result (realistic photos), and complications with probabilities

•Registered nurse – 30 min: preparing for surgery, postoperative recovery, and how to navigate the health-care system

**Figure 3:**
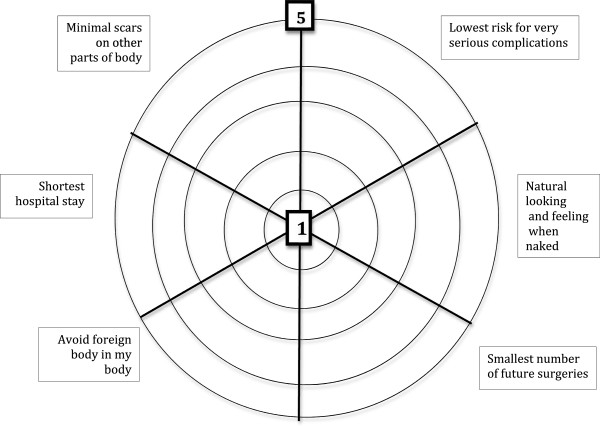
**Values clarification exercise.** The last component of the Pre-Consultation Educational Group Intervention is the values clarification exercise. Using an interactive example, the purpose of the values clarification exercise is explained, and patients are invited to consider their personal values and complete the exercise. This is brought to the surgical consultation to incorporate patient values into the shared decision-making process.

•Social worker (SW) – 30 min: values clarification exercise (Figure 3)

•Breast reconstruction patient volunteer: 30 min: questions and answers about her personal experience

Patients in the experimental and control group will receive usual care. At the UHN, all patients are asked to read a breast reconstruction information booklet and visit the website developed by the group prior to their consultation with the plastic surgeons. They are then booked for a 45-min consultation with a consultant plastic surgeon and a surgical resident or fellow. A thorough history and physical exam are performed, and specific information regarding reconstruction options, advantages, and complications is provided, followed by a discussion about patient preferences. In addition, all patients are referred to speak with a volunteer patient at a later date if desired. Appointments will be scheduled in batches to prevent contamination between those randomized to the experimental and control groups.

### Outcome measures

#### Pilot and feasibility outcome measures

*Feasibility and acceptability of randomization, treatment allocation, and data-collection procedures:* Recruitment and attrition rates will be recorded. We will measure the proportion of participants that submits *complete* primary and secondary outcome measures and baseline questionnaires. *Treatment Implementation and fidelity:* We will record the proportion of participants who received their randomly allocated treatment assignment to monitor participant adherence to treatment allocation. *Acceptability of interventions, assessment of primary outcome, and refinement of the educational group intervention content and delivery:* A subgroup of participants allocated to both the experimental and usual care groups will be asked to participate in a brief qualitative telephone interview. Purposeful sampling will be used to recruit five patients from each group to achieve data saturation and variability [31]. Telephone interviews will be conducted by a social worker trained in qualitative methods after the decision regarding breast reconstruction has been made, which we consider to be when a patient signs the surgical consent. All participants randomized to the workshop will additionally be asked to complete a written survey for evaluation of the intervention immediately after participation in the workshop.

#### Trial outcome measures will be divided into three broad categories

*Decision measures* will include decision self-efficacy [21], decision conflict score [32], decision preference, and decision choice [33]. These three instruments will be given at T0 (baseline) and T1, after the surgical consultation with the plastic surgeon.

1) The decision self-efficacy (DSE) scale is a prospectively designed instrument to evaluate patient self-confidence in decision-making, including shared decision-making [21]. It has been validated among women facing treatment decisions for osteoporosis [21] and used in cancer patients [22]. Psychometric evaluation has shown high levels of internal consistency (Cronbach’s alpha 0.90). Decision self-efficacy is correlated with decision conflict subscales of feeling informed (*r* = 0.47) and supported (*r* = 0.45) [21]. This instrument has never been tested in the breast cancer or breast reconstruction population.

2) The decision conflict scale measures personal perceptions of uncertainty in choosing options and has been demonstrated to be valid and responsive to change [32]. The decisional conflict scale is a 16-item 5-response instrument that reports a score from 0 – 100 with higher scores indicating more conflict (items are summed, divided by 16 and multiplied by 25) [26,34].

3) Decision Preference and Decision Choice has been used as a primary and secondary outcome in studies of decision support interventions in cancer patients. It demonstrates good test-retest reliability (test-retest coefficient > 0.90) and is sensitive to change when measured before and after an intervention [33].

*Patient reported outcomes (PRO)* will be measured using the perceived involvement in care scale [35] (PICS) and satisfaction with information subscale of BREAST-Q [36-39].

1) PICS is a measure of patient perception of involvement with her care, and has seven 5-point Likert scale items that assess the extent to which the patient asked questions, offered opinions, and expressed concerns when meeting with the surgeon [35].

2) The BREAST-Q is a procedure-specific and validated PRO that measures Hr-QOL and patient satisfaction with PMBR [36-39]. The “Satisfaction with Information” Subscale specifically measures patient satisfaction with the preoperative information and care provided by the plastic surgeon and other members of the medical team. There are 15 items that use a four-level Likert scale response format; the score is transformed on a scale of 0 to 100 with higher scores indicating greater satisfaction [36-39].

*Service outcomes* will be measured using the uptake rate of breast reconstruction, consultation length, number of consultations until a decision is made (consent signed), the physician satisfaction scale [40], and a breast reconstruction knowledge test [41].

### Baseline measures

Patient demographic and clinical characteristics, baseline psychosocial measures such as the Spielberger State-Trait Anxiety Inventory (STAI) [42], a social support survey [43], and preference for decision control [44] will be captured on our routine two-page patient intake form after consent but prior to randomization and allocation (T0, Figure 1). Baseline covariates that will be documented include demographic variables, including age, marital status, ethnicity, household annual income, highest level of education, urban vs. nonurban place of residence, and employment status, and clinical covariates, including study site, timing of the reconstruction (immediate vs. delayed), previous stage of breast cancer, prior receipt of radiation, prior receipt of chemotherapy, and presence of chronic illness.

STAI is a 20-item measure of anxiety, and its scores range from 20–80, with a higher score indicating greater anxiety [42]. The STAI trait subscale will be measured at baseline, and the state subscale will be measured at baseline and T1. The state subscale is a sensitive indicator of changes in transitory anxiety in behavior modification programs or with experimental procedures.

The Medical Outcomes Study Social Support Survey has a series of 18 questions that measure four domains of social support (emotional, tangible, affectionate, and social interactions). Responses range from 1 (none of the time) to 5 (all the time). The items in each domain were summed and then transformed to yield scores ranging from 0 to 100. Higher scores indicate more support [43].

Preference for Decision Control is assessed using a validated question from previous studies in cancer patients where patients indicate whether they want to play an active, passive, or collaborative role with their physician when making a treatment decision [35].

### Analysis

Analysis of primary and secondary outcomes from the main trial will be summarized using measures of central tendency and dispersion. This pilot study will provide important information regarding the treatment effect estimate and variability (standard deviation) to supplement what has been reported in observational studies and will be used to guide the design and sample size calculation for the main trial. Because this is a pilot study, we do not propose to undertake formal hypothesis tests of the primary or secondary endpoints for the definitive trial.

### Qualitative analysis

Thematic analysis [45] will be used to extract emerging themes and variations. Concurrent data collection and analysis will be undertaken using multi-layered reading and constant comparison [46]. N-vivo 10.0 software will be used for data management. An audit trail, detailed field notes, and memos will be maintained throughout the research process.

### Sample size and power

Sample size for the pilot study is determined based on the recommended sample size of approximately 40 participants for a pilot study [47]. Approximately 20 patients are referred for breast reconstruction per surgeon per month. Workshops and clinics have the capacity for five and ten patients per session, respectively. A conservative estimate of 50-70% recruitment would allow us to recruit our pilot sample size over 6 months across four clinics.

### Interpretation of pilot results

Feasibility targets include ≥ 60% recruitment, ≥ 80% retention after randomized treatment assignment, and ≥ 80% completion of primary outcome measure. Success will also be measured based on high treatment fidelity measured through observation of the intervention and participant qualitative report. To inform our decision for the primary outcome for a definitive RCT, the most important outcome measure for patients will be solicited and ascertained through qualitative methods.

## Discussion

To reduce unnecessary variation in treatment and improve quality of cancer care among breast cancer patients, there has been a growing interest in engaging patients through the decision-making process [48,49]. Particularly for breast reconstruction there is a paucity of studies designed to address patients’ unique informational and supportive-care needs, and there has been a call to action to develop and implement interventions that enhance patient empowerment [50]. Only one RCT has designed and implemented a decision support intervention for patients considering breast reconstruction [51]. While patients assigned to the pre-consultation computer-based decision support intervention demonstrated greater knowledge scores, outcomes related to shared decision-making or decision quality were not assessed using validated instruments [51]. Retrospective observational studies from single institutions suggest that well-informed patients already participate in the decision whether or not to undergo breast reconstruction [12,41], but interventions can improve involvement and satisfaction for the decision regarding the type of reconstruction (implant-based or autologous tissue reconstruction) among women who decided to have breast reconstruction [52]. However, inferences regarding the utility of decision-support interventions for breast reconstruction patients from non-randomized and particularly retrospective studies must be made with caution. Furthermore, no study has examined the potential of group workshops to enhance patient engagement and decision quality; educational interventions delivered in a group setting have been demonstrated to be an effective tool to address informational and psychosocial needs for breast cancer survivors [30]. Therefore, we designed a novel intervention to address identified gaps in care to promote shared decision-making and improve the quality of patient decisions surrounding breast reconstruction.

Because the intervention occurs *before* the initial surgical consultation (necessitating telephone recruitment) and is delivered using a *novel format* in a group setting, we felt it would be most appropriate to first undertake a pilot study prior to a definitive trial. Our pilot study seeks to identify (1) the feasibility, acceptability, and design of a definitive RCT and (2) the optimal content and delivery of our proposed educational group intervention. Results from our pilot study will be used in the following two ways: (1) to validate the design of the intervention and revise content and delivery to meet the needs of the end-users; (2) to inform the final design and sample size estimation for a definitive RCT.

## Trial status

The pre-consultation educational group intervention pilot trial began recruitment in January 2013. Thirty patients have been recruited to date (18 May 2013), of whom 15 have been randomized to one of three decision support workshops. Once all participants have been recruited and enrolled, we will complete qualitative interviews among a purposeful sample of voluntary participants. Recruitment is ongoing, and the trial is scheduled to be completed by the end of this academic year (June 2013).

## Competing interests

The authors declare they have no competing interests.

## Authors’ contributions

Conception and study design (TZ, JP, NB, JJ, NC, KM); recruitment of participants (TZ, SH, AO, NC); development and implementation of workshop (JP, TZ, NC, TC, ES, JJ, SH, AO); drafting of manuscript (JP, TZ); critical review of manuscript (all authors). All authors read and approved the final manuscript.

## Research support

This research was supported by the Physician Services Incorporated Foundation-Resident Grant awarded to Dr. Jennica Platt under the supervision of Dr. Toni Zhong. The opinions reported in this article are those of the authors and are independent from the funding source.
